# 7,11,15,28-Tetra­kis[(2-formyl­phen­oxy)methyl]-1,21,23,25-tetra­methyl­resorcin[4]arene cavitand ethyl acetate clathrate at 173 K

**DOI:** 10.1107/S1600536809007582

**Published:** 2009-03-06

**Authors:** Michael G. Mc Kay, Holger B. Friedrich, R. Alan Howie, Glenn E. M. Maguire

**Affiliations:** a School of Chemistry, University of KwaZulu-Natal, Durban, 4000, South Africa; b Department of Chemistry, University of Aberdeen, Aberdeen AB24 3UE, Scotland

## Abstract

The title compound, C_68_H_56_O_16_, was synthesized as a novel synthetic inter­mediate towards deeper and more elaborate resorcin[4]arene cavitands. The structure is the first reported example of a resorcin[4]arene cavitand bearing aromatic aldehyde functional groups at the extra-annular rim of the mol­ecule. The 2-formyl­phen­oxy residues are found to assume two different orientations above the mol­ecular cavity. One half of the resorcin[4]arene cavitand mol­ecule appears in the asymmetric unit; the complete resorcin[4]arene cavitand structure was generated across a mirror plane. In addition, a highly disordered ethyl acetate solvent mol­ecule is present within the mol­ecular cavity.

## Related literature

For literature pertaining to the preparation of precursors to the reported compound, see: Middel *et al.* (2001 ▶); Sorrell & Pigge (1993 ▶). For related literature on synthetic analogues and other precursors which illustrate the host capabilities of resorcin[4]arene cavitand molecules, see:  Friedrich *et al.* (2007 ▶); Mc Kay *et al.* (2007 ▶, 2008 ▶). For the implemetation of the SQUEEZE function in *PLATON*, see: Tam *et al.* (2005 ▶). 
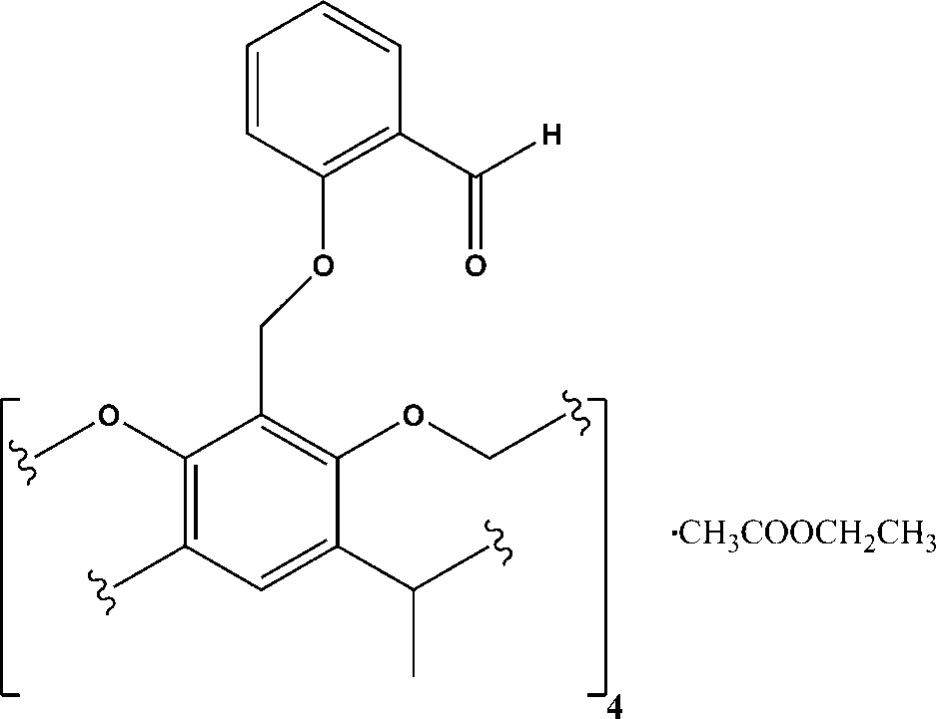


## Experimental

### 

#### Crystal data

C_68_H_56_O_16_
*M* *_r_* = 1183.17
Monoclinic, 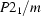

*a* = 11.9228 (7) Å
*b* = 23.2806 (15) Å
*c* = 12.2320 (7) Å
β = 117.005 (3)°
*V* = 3025.0 (3) Å^3^
*Z* = 2
Mo *K*α radiation
μ = 0.09 mm^−1^
*T* = 173 K
0.37 × 0.34 × 0.26 mm

#### Data collection

Bruker APEXII CCD area-detector diffractometer
Absorption correction: integration (*SAINT-NT*; Bruker, 2005 ▶) *T*_min_ = 0.967, *T*_max_ = 0.977
24858 measured reflections
5470 independent reflections
3556 reflections with *I* > 2σ(*I*)
*R*_int_ = 0.079

#### Refinement

*R*[*F* ^2^ > 2σ(*F* ^2^)] = 0.074
*wR*(*F* ^2^) = 0.230
*S* = 1.11
5470 reflections
417 parameters
21 restraints
H-atom parameters constrained
Δρ_max_ = 0.64 e Å^−3^
Δρ_min_ = −0.48 e Å^−3^

### 

Data collection: *APEX2* (Bruker, 2005 ▶); cell refinement: *SAINT-NT* (Bruker, 2005 ▶); data reduction: *SAINT-NT*; program(s) used to solve structure: *SHELXTL* (Sheldrick, 2008 ▶); program(s) used to refine structure: *SHELXL97* (Sheldrick, 2008 ▶); molecular graphics: *ORTEP-3* (Farrugia, 1997 ▶); software used to prepare material for publication: *ORTEP-3 for Windows* (Farrugia, 1997 ▶) and *PLATON* (Spek, 2009 ▶).

## Supplementary Material

Crystal structure: contains datablocks I, New_Global_Publ_Block. DOI: 10.1107/S1600536809007582/fl2233sup1.cif

Structure factors: contains datablocks I. DOI: 10.1107/S1600536809007582/fl2233Isup2.hkl

Additional supplementary materials:  crystallographic information; 3D view; checkCIF report

## Figures and Tables

**Fig. 1.**
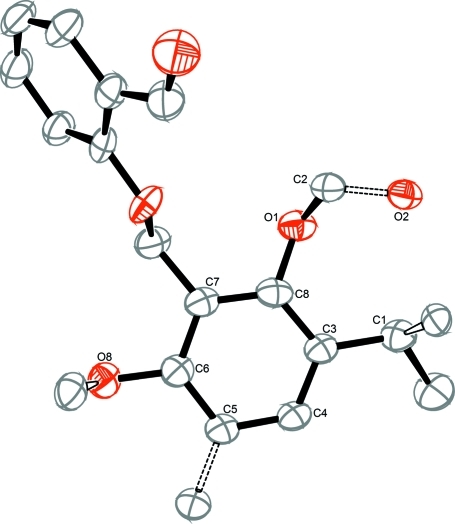
A view of one component of the cyclic tetramer. Displacement ellipsoids are drawn at the 30% probability level and H atoms are shown as spheres of arbitrary radii. Dashed bonds indicate links to the neighbouring monomer units. Selected atoms are labelled.

**Fig. 2.**
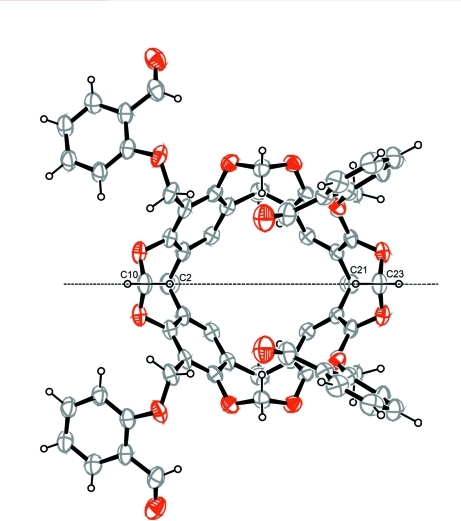
The molecular structure of the title compound, as viewed from above the molecular cavity. Displacement ellipsoids are drawn at the 30% probability level. The relative orientations of the 2-formylphenoxy residues are evident. The dashed line indicates the mirror plane, which passes through C2, C10, and C21–23.

**Fig. 3.**
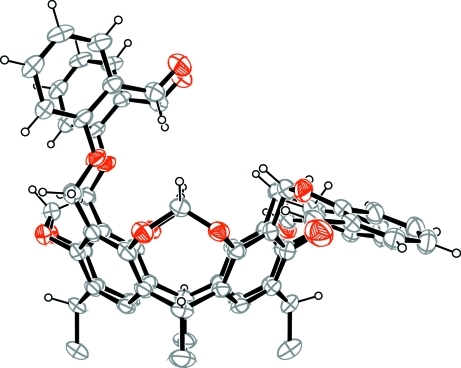
The molecular structure of the title compound viewed from side-on. Displacement ellipsoids are drawn at the 30% probability level and H atoms, where shown, are drawn as spheres of arbitrary radii. The relative orientations of the 2-formylphenoxy functional groups are illustrated.

## supplementary crystallographic information

### Comment

In the title compound (Scheme 1) the [4]arene moiety is a cyclic tetramer. The labelling scheme for one of the monomers (Fig.1) extends over the whole molecule. Dimensions are available in the archived CIF. Additionally, the bond lengths and bond angles present in the asymmetric unit fall within the normal ranges and are not discussed further.

We have previously reported a number of resorcin[4]arene structures of the synthetic precursors to the title compound. However, the novel title compound was synthesized in a Williamson-type ether synthesis using a bromomethyl resorcin[4]arene cavitand precursor. For literature pertaining to the preparation of precursors to the reported compound, see: Middel *et al.* (2001); Sorrell & Pigge (1993). For related literature on synthetic analogues and other precursors which illustrate the host capabilities of resorcin[4]arene cavitand molecules, see: Friedrich *et al.* (2007); Mc Kay *et al.* (2007, 2008). For the implemetation of the SQUEEZE function in *PLATON*, see: Tam *et al.* (2005).

The title compound exhibits two different orientations of the 2-formylphenoxy residues, which are present above the molecular cavity. Two adjacent residues appear upright, while the remaining two appear splayed, in an orientation almost perpendicular to the first two residues. This relative orientation is illustrated in Fig. 2 (above the molecular cavity) and Fig. 3 (side view). Additionally, the asymmetric unit consists of a half of the title compound with the complete molecule being generated by  a crystallographic mirror plane with atoms C1, C2, C10 and C21–23 in special positions on the mirror plane. This plane is indicated by a dashed line in Fig. 2.

Our previous resorcin[4]arene structures show the presence of residual solvent molecules present within the confines of the molecular cavity. The title compound exhibited the presence of ethyl acetate occupying the molecular cavity. However, the ethyl acetate was of a highly disordered nature. Therefore, in the final refinement model, the electron density related to the disordered ethyl acetate molecule was removed by using the SQUEEZE function of *PLATON* (Spek, 2009). This resulted in an improved refinement model, and eliminated the ill-effects of the disorder upon the refinement. The contribution of the ethyl acetate molecule was not included in the molecular formula, but is detailed in the SQUEEZE results which are appended to the CIF text. This further accounts for the discrepancies seen in the calculated and reported parameters of molecular weight, density and absorption coefficient.

### Experimental

To a stirring solution of salicylaldehyde (1.01 g, 8.30 mmol) in dry THF (70 ml) under a nitrogen atmosphere, NaH (60% suspension in mineral oil, 0.33 g, 8.30 mmol) was added. To the resulting bright yellow solution, bromomethyl cavitand (I) (1.00 g, 1.04 mmol) was added as a solution in dry THF (10 ml), dropwise over 30 minutes. The solution was refluxed for 4 days; TLC showed mono-, di-, tri- and tetra-substituted products. Over this time, the solution became grey in colour. Once cooled to room temperature, the solution was concentrated *in vacuo*. The products were chromatographed on silica gel using a mobile phase of 3:2 hexane-ethyl acetate. Fractions of 12 ml were collected, combining all fractions containing the desired tetra-substituted product (*R*_f_ =0.37) after separation. The purified product was concentrated using a rotary evaporator to yield an off-white solid; this was then stirred in methanol overnight. After filtration, the product was collected and stirred overnight in hexane to remove residual aldehyde, before being filtered and collected to yield the title compound as a white solid. (0.35 g, 28%), mp 400 K. ^1^H NMR [CDCl_3_, 300 MHz]: δ = 1.88 (d, *J* = 7.6 Hz, 12 H, C*H*_3_), 4.57 (d, *J*= 6.9 Hz, 4 H, inner of OC*H*_2_O),4.96 (s, 8 H, ArOC*H*_2_Ar),5.05 (q, *J* = 7.1 Hz, 4 H, C*H*CH_3_), 5.82 (d, *J* = 6.8 Hz, 4 H, outer of OC*H*_2_O), 7.04 (t, *J* = 7.1 Hz, 4 H, Ar *H*), 7.14 (d, *J* = 8.5 Hz,4 H, Ar *H*), 7.40 (s, 4 H, cavitand Ar*H*), 7.53 (t, *J* = 7.0 Hz, 4 H, Ar *H*),7.73 (d, *J* = 7.6 Hz, 4 H, Ar *H*), 10.18 (s, 4 H, Ar C*H*O). ^13^CNMR [CDCl_3_, 75 MHz]: δ = 189.83,160.93, 154.04, 139.09, 135.88, 129.70, 125.56, 121.77, 121.43, 114.18, 100.00,62.07, 31.22, 16.14. Anal Calcd for C_68_H_56_O_16_(1129.17): C 72.33, H 4.99. Found: C 72.58, H 5.08.

Crystals suitable for X-ray crystallography were grown by slow evaporation of a solution of the title compound in 1:1 ethyl acetate:hexane, at ambient temperature.

### Refinement

The crystal structure was solved by direct methods using *SHELXTL* (Sheldrick, 2008). Non-hydrogen atoms were first refined isotropically followed by anisotropic refinement by full matrix least-squares calculations based on F2 using *SHELXTL*. Hydrogen atoms, first located in the difference map, were positioned geometrically and allowed to ride on their respective parent atoms, with C—H bond lengths of 1.00 (CH), 0.99 (CH_2_), or 0.98 (CH_3_). They were then refined with a riding model with *U*_iso_(H) = 1.5*U*_eq_(CH_3_) and *U*_iso_(H) = 1.2*U*_eq_(*X*) for *X* = CH or CH_2_.

One of the 2-formylphenoxy residues is disordered. The residue was refined over two positions with the final occupancies being 0.789 (4) for atoms O5, O39 and C32–38, and 0.211 (4) for atoms O5A, O39A and C32A-38 A. The largest residual electron density peak of 0.64 e/Å^3^ is 0.92 Å from H38A.

### Crystal data

**Table:**

C_68_H_56_O_16_	*Z* = 2
*M**_r_* = 1183.17	*F*(000) = 1238
Monoclinic, *P*2_1_/*m*	*D*_x_ = 1.261 Mg m^−^^3^
Hall symbol: -P 2yb	Melting point: 400 K
*a* = 11.9228 (7) Å	Mo *K*α radiation, λ = 0.71073 Å
*b* = 23.2806 (15) Å	µ = 0.09 mm^−^^1^
*c* = 12.2320 (7) Å	*T* = 173 K
β = 117.005 (3)°	Block, colourless
*V* = 3025.0 (3) Å^3^	0.37 × 0.34 × 0.26 mm

### Data collection

**Table:**

Bruker APEXII CCD area-detector diffractometer	5470 independent reflections
Radiation source: fine-focus sealed tube	3556 reflections with *I* > 2σ(*I*)
graphite	*R*_int_ = 0.079
phi and ω scans	θ_max_ = 25.0°, θ_min_ = 1.8°
Absorption correction: integration (*SAINT-NT*; Bruker, 2005)	*h* = −14→13
*T*_min_ = 0.967, *T*_max_ = 0.977	*k* = −27→27
24858 measured reflections	*l* = −14→14

### Refinement

**Table:**

Refinement on *F*^2^	Primary atom site location: structure-invariant direct methods
Least-squares matrix: full	Secondary atom site location: difference Fourier map
*R*[*F*^2^ > 2σ(*F*^2^)] = 0.074	Hydrogen site location: inferred from neighbouring sites
*wR*(*F*^2^) = 0.230	H-atom parameters constrained
*S* = 1.11	*w* = 1/[σ^2^(*F*_o_^2^) + (0.1125*P*)^2^ + 0.9365*P*] where *P* = (*F*_o_^2^ + 2*F*_c_^2^)/3
5470 reflections	(Δ/σ)_max_ < 0.001
417 parameters	Δρ_max_ = 0.64 e Å^−^^3^
21 restraints	Δρ_min_ = −0.48 e Å^−^^3^

### Special details

**Table:**

Geometry. All e.s.d.'s (except the e.s.d. in the dihedral angle between two l.s. planes) are estimated using the full covariance matrix. The cell e.s.d.'s are taken into account individually in the estimation of e.s.d.'s in distances, angles and torsion angles; correlations between e.s.d.'s in cell parameters are only used when they are defined by crystal symmetry. An approximate (isotropic) treatment of cell e.s.d.'s is used for estimating e.s.d.'s involving l.s. planes.
Refinement. Refinement of *F*^2^ against ALL reflections. The weighted *R*-factor *wR* and goodness of fit *S* are based on *F*^2^, conventional *R*-factors *R* are based on *F*, with *F* set to zero for negative *F*^2^. The threshold expression of *F*^2^ > σ(*F*^2^) is used only for calculating *R*-factors(gt) *etc*. and is not relevant to the choice of reflections for refinement. *R*-factors based on *F*^2^ are statistically about twice as large as those based on *F*, and *R*- factors based on ALL data will be even larger.

### Fractional atomic coordinates and isotropic or equivalent isotropic displacement parameters (Å^2^)

**Table:**

	*x*	*y*	*z*	*U*_iso_*/*U*_eq_	Occ. (<1)
O1	−0.0864 (2)	0.19966 (13)	0.2854 (2)	0.0789 (7)	
O2	0.0149 (2)	0.04856 (13)	0.4185 (2)	0.0841 (8)	
O3	0.2250 (2)	0.05596 (10)	0.3626 (2)	0.0684 (6)	
O4	0.4399 (2)	0.05649 (11)	0.4109 (2)	0.0724 (7)	
O5	0.6322 (6)	0.1273 (4)	0.6538 (3)	0.0687 (14)	0.789 (4)
O6	0.7467 (2)	0.19955 (12)	0.4748 (2)	0.0717 (7)	
C1	−0.0852 (6)	0.2500	−0.0474 (5)	0.108 (2)	
H1A	−0.1344	0.2156	−0.0867	0.162*	0.50
H1B	−0.0074	0.2500	−0.0560	0.162*	
H1C	−0.1344	0.2844	−0.0867	0.162*	0.50
C2	−0.0529 (5)	0.2500	0.0879 (4)	0.0759 (15)	
H2	−0.1348	0.2500	0.0921	0.091*	
C3	0.0185 (3)	0.19689 (17)	0.1568 (3)	0.0638 (9)	
C4	0.1057 (3)	0.16939 (15)	0.1293 (3)	0.0612 (9)	
H4	0.1182	0.1834	0.0627	0.073*	
C5	0.1755 (3)	0.12235 (15)	0.1947 (3)	0.0595 (8)	
C6	0.1587 (3)	0.10305 (15)	0.2943 (3)	0.0618 (8)	
C7	0.0711 (3)	0.12873 (17)	0.3245 (3)	0.0651 (9)	
C8	0.0030 (3)	0.17497 (17)	0.2554 (3)	0.0667 (9)	
C10	−0.0484 (5)	0.2500	0.3569 (5)	0.0784 (15)	
H10A	−0.0846	0.2500	0.4155	0.094*	
H10B	0.0444	0.2500	0.4051	0.094*	
C11	0.0564 (3)	0.10681 (18)	0.4335 (3)	0.0758 (10)	
H11A	0.1382	0.1099	0.5081	0.091*	
H11B	−0.0051	0.1312	0.4456	0.091*	
C12	0.3502 (3)	0.06610 (17)	0.4547 (3)	0.0679 (9)	
H12A	0.3574	0.1063	0.4837	0.081*	
H12B	0.3687	0.0405	0.5254	0.081*	
C13	0.4750 (3)	0.10434 (15)	0.3670 (3)	0.0624 (9)	
C14	0.4015 (3)	0.12373 (14)	0.2479 (3)	0.0577 (8)	
C15	0.2761 (4)	0.09540 (16)	0.1678 (3)	0.0698 (9)	
H15	0.2839	0.0545	0.1954	0.084*	
C16	0.2419 (5)	0.0937 (2)	0.0314 (4)	0.0927 (13)	
H16A	0.3103	0.0754	0.0205	0.139*	
H16B	0.2292	0.1329	−0.0010	0.139*	
H16C	0.1642	0.0716	−0.0128	0.139*	
C17	0.4452 (3)	0.17050 (14)	0.2087 (3)	0.0576 (8)	
H17	0.3953	0.1847	0.1281	0.069*	
C18	0.5590 (3)	0.19770 (14)	0.2820 (3)	0.0560 (8)	
C19	0.6293 (3)	0.17645 (16)	0.3996 (3)	0.0618 (9)	
C20	0.5909 (3)	0.12927 (15)	0.4442 (3)	0.0610 (8)	
C21	0.6021 (5)	0.2500	0.2383 (4)	0.0624 (12)	
H21	0.6962	0.2500	0.2838	0.075*	
C22	0.5690 (6)	0.2500	0.1040 (5)	0.0819 (16)	
H22A	0.6040	0.2156	0.0847	0.123*	0.50
H22B	0.6040	0.2844	0.0847	0.123*	0.50
H22C	0.4774	0.2500	0.0551	0.123*	
C23	0.7479 (5)	0.2500	0.5390 (5)	0.0751 (15)	
H23A	0.6736	0.2500	0.5549	0.090*	
H23B	0.8242	0.2500	0.6193	0.090*	
C24	−0.1063 (3)	0.03514 (18)	0.3420 (3)	0.0698 (10)	
C25	−0.1265 (3)	−0.02235 (19)	0.3029 (3)	0.0724 (10)	
C26	−0.2490 (4)	−0.04163 (19)	0.2342 (3)	0.0790 (11)	
H26	−0.2640	−0.0807	0.2096	0.095*	
C27	−0.3492 (4)	−0.0044 (2)	0.2014 (4)	0.0877 (12)	
H27	−0.4331	−0.0175	0.1540	0.105*	
C28	−0.3259 (4)	0.0512 (2)	0.2380 (4)	0.0853 (12)	
H28	−0.3951	0.0767	0.2145	0.102*	
C29	−0.2063 (3)	0.07220 (19)	0.3078 (3)	0.0759 (10)	
H29	−0.1932	0.1114	0.3316	0.091*	
C30	−0.0200 (4)	−0.0605 (2)	0.3320 (4)	0.0855 (12)	
H30	0.0624	−0.0453	0.3772	0.103*	
O31	−0.0307 (3)	−0.11081 (18)	0.3016 (3)	0.1079 (10)	
C32	0.6930 (10)	0.1091 (4)	0.7722 (4)	0.0610 (12)	0.789 (4)
C33	0.6320 (4)	0.1178 (2)	0.8485 (4)	0.0651 (12)	0.789 (4)
C34	0.6952 (5)	0.1003 (2)	0.9714 (4)	0.0819 (15)	0.789 (4)
H34	0.6588	0.1078	1.0247	0.098*	0.789 (4)
C35	0.8071 (9)	0.0730 (3)	1.0151 (6)	0.086 (3)	0.789 (4)
H35	0.8481	0.0609	1.0984	0.103*	0.789 (4)
C36	0.8608 (5)	0.0627 (3)	0.9406 (6)	0.084 (2)	0.789 (4)
H36	0.9401	0.0438	0.9729	0.101*	0.789 (4)
C37	0.8035 (4)	0.0789 (2)	0.8188 (5)	0.0721 (17)	0.789 (4)
H37	0.8408	0.0691	0.7672	0.086*	0.789 (4)
C38	0.5084 (5)	0.1450 (2)	0.8038 (4)	0.0801 (14)	0.789 (4)
H38	0.4685	0.1578	0.7210	0.096*	0.789 (4)
O39	0.4540 (4)	0.1522 (2)	0.8650 (4)	0.1058 (14)	0.789 (4)
O5A	0.615 (3)	0.1252 (18)	0.6415 (18)	0.076 (2)*	0.211 (4)
C32A	0.687 (5)	0.1056 (19)	0.7573 (19)	0.076 (2)*	0.211 (4)
C33A	0.6917 (17)	0.1491 (7)	0.8415 (13)	0.076 (2)*	0.211 (4)
C34A	0.7500 (17)	0.1340 (8)	0.9670 (14)	0.076 (2)*	0.211 (4)
H34A	0.7415	0.1584	1.0251	0.091*	0.211 (4)
C35A	0.817 (4)	0.0855 (13)	1.005 (2)	0.076 (2)*	0.211 (4)
H35A	0.8562	0.0760	1.0897	0.091*	0.211 (4)
C36A	0.829 (3)	0.0498 (11)	0.924 (2)	0.076 (2)*	0.211 (4)
H36A	0.8768	0.0154	0.9523	0.091*	0.211 (4)
C37A	0.774 (2)	0.0625 (10)	0.800 (2)	0.076 (2)*	0.211 (4)
H37A	0.7967	0.0419	0.7455	0.091*	0.211 (4)
C38A	0.6207 (16)	0.2018 (7)	0.7993 (15)	0.076 (2)*	0.211 (4)
H38A	0.5835	0.2110	0.7145	0.091*	0.211 (4)
O39A	0.6082 (10)	0.2326 (4)	0.8661 (10)	0.076 (2)*	0.211 (4)
C40	0.6725 (3)	0.10524 (18)	0.5674 (3)	0.0732 (10)	
H40A	0.6669	0.0628	0.5648	0.088*	
H40B	0.7613	0.1162	0.5932	0.088*	

### Atomic displacement parameters (Å^2^)

**Table:**

	*U*^11^	*U*^22^	*U*^33^	*U*^12^	*U*^13^	*U*^23^
O1	0.0513 (13)	0.109 (2)	0.0848 (17)	−0.0066 (13)	0.0381 (12)	−0.0015 (15)
O2	0.0592 (14)	0.110 (2)	0.0818 (17)	−0.0194 (14)	0.0305 (13)	0.0159 (15)
O3	0.0759 (15)	0.0721 (15)	0.0636 (13)	−0.0104 (12)	0.0372 (12)	0.0058 (12)
O4	0.0813 (15)	0.0676 (15)	0.0778 (15)	0.0124 (12)	0.0445 (13)	0.0155 (12)
O5	0.060 (3)	0.085 (3)	0.0437 (17)	0.024 (3)	0.0084 (17)	0.0045 (18)
O6	0.0500 (12)	0.0983 (19)	0.0715 (14)	0.0077 (12)	0.0317 (11)	0.0066 (14)
C1	0.093 (4)	0.131 (6)	0.064 (3)	0.000	0.004 (3)	0.000
C2	0.051 (3)	0.105 (4)	0.055 (3)	0.000	0.009 (2)	0.000
C3	0.0461 (16)	0.089 (3)	0.0461 (16)	−0.0079 (16)	0.0124 (14)	−0.0023 (16)
C4	0.0585 (18)	0.083 (2)	0.0351 (15)	−0.0130 (17)	0.0152 (13)	−0.0064 (15)
C5	0.0605 (18)	0.075 (2)	0.0435 (16)	−0.0131 (17)	0.0243 (14)	−0.0125 (16)
C6	0.0603 (18)	0.072 (2)	0.0500 (17)	−0.0151 (16)	0.0220 (15)	−0.0022 (16)
C7	0.0482 (16)	0.090 (3)	0.0596 (19)	−0.0192 (17)	0.0264 (15)	−0.0011 (18)
C8	0.0468 (17)	0.090 (3)	0.0603 (19)	−0.0107 (17)	0.0215 (15)	−0.0037 (19)
C10	0.061 (3)	0.110 (5)	0.079 (3)	0.000	0.045 (3)	0.000
C11	0.065 (2)	0.099 (3)	0.069 (2)	−0.019 (2)	0.0360 (18)	0.006 (2)
C12	0.071 (2)	0.078 (2)	0.061 (2)	−0.0017 (18)	0.0345 (17)	0.0129 (18)
C13	0.072 (2)	0.062 (2)	0.070 (2)	0.0124 (17)	0.0477 (18)	0.0089 (17)
C14	0.0650 (18)	0.067 (2)	0.0520 (17)	0.0070 (16)	0.0365 (15)	−0.0007 (15)
C15	0.088 (2)	0.072 (2)	0.0560 (19)	−0.0051 (19)	0.0382 (18)	−0.0074 (17)
C16	0.117 (3)	0.108 (3)	0.061 (2)	−0.013 (3)	0.048 (2)	−0.018 (2)
C17	0.0688 (19)	0.072 (2)	0.0451 (16)	0.0073 (16)	0.0370 (15)	0.0013 (15)
C18	0.0601 (18)	0.067 (2)	0.0564 (17)	0.0081 (15)	0.0404 (15)	0.0012 (15)
C19	0.0542 (17)	0.082 (2)	0.0614 (19)	0.0096 (16)	0.0366 (16)	0.0044 (17)
C20	0.0608 (19)	0.071 (2)	0.0608 (18)	0.0176 (16)	0.0364 (16)	0.0126 (16)
C21	0.067 (3)	0.082 (3)	0.056 (2)	0.000	0.044 (2)	0.000
C22	0.106 (4)	0.091 (4)	0.079 (3)	0.000	0.068 (3)	0.000
C23	0.054 (3)	0.111 (5)	0.062 (3)	0.000	0.028 (2)	0.000
C24	0.066 (2)	0.095 (3)	0.062 (2)	−0.0045 (19)	0.0404 (17)	0.0028 (19)
C25	0.064 (2)	0.108 (3)	0.060 (2)	0.000 (2)	0.0416 (17)	−0.004 (2)
C26	0.076 (2)	0.097 (3)	0.069 (2)	−0.002 (2)	0.0376 (19)	−0.022 (2)
C27	0.057 (2)	0.115 (4)	0.088 (3)	−0.005 (2)	0.0306 (19)	−0.026 (3)
C28	0.065 (2)	0.100 (3)	0.101 (3)	−0.005 (2)	0.046 (2)	−0.027 (2)
C29	0.062 (2)	0.093 (3)	0.083 (2)	−0.0054 (19)	0.0419 (19)	−0.017 (2)
C30	0.082 (3)	0.113 (4)	0.080 (3)	0.017 (3)	0.052 (2)	0.007 (3)
O31	0.112 (2)	0.121 (3)	0.107 (2)	0.028 (2)	0.064 (2)	0.005 (2)
C32	0.054 (2)	0.064 (3)	0.046 (2)	−0.002 (2)	0.006 (2)	−0.011 (2)
C33	0.067 (3)	0.069 (3)	0.051 (2)	−0.005 (2)	0.020 (2)	−0.011 (2)
C34	0.092 (4)	0.092 (4)	0.046 (2)	0.003 (3)	0.018 (2)	−0.003 (2)
C35	0.099 (5)	0.089 (5)	0.042 (3)	−0.005 (4)	0.008 (3)	0.005 (3)
C36	0.066 (4)	0.093 (5)	0.067 (3)	0.008 (3)	0.006 (3)	0.007 (3)
C37	0.054 (3)	0.095 (4)	0.060 (3)	0.009 (3)	0.019 (2)	0.010 (3)
C38	0.073 (3)	0.105 (4)	0.068 (3)	−0.005 (3)	0.037 (2)	−0.011 (3)
O39	0.102 (3)	0.140 (4)	0.095 (3)	0.003 (2)	0.062 (2)	−0.012 (2)
C40	0.0610 (19)	0.094 (3)	0.070 (2)	0.0192 (18)	0.0347 (17)	0.021 (2)

### Geometric parameters (Å, °)

**Table:**

O1—C8	1.399 (4)	C22—H22B	0.9800
O1—C10	1.409 (4)	C22—H22C	0.9800
O2—C24	1.354 (4)	C23—O6^i^	1.410 (4)
O2—C11	1.426 (5)	C23—H23A	0.9900
O3—C6	1.387 (4)	C23—H23B	0.9900
O3—C12	1.424 (4)	C24—C29	1.374 (5)
O4—C13	1.382 (4)	C24—C25	1.405 (6)
O4—C12	1.414 (4)	C25—C26	1.388 (5)
O6—C19	1.387 (4)	C25—C30	1.455 (6)
O6—C23	1.410 (4)	C26—C27	1.379 (6)
C1—C2	1.519 (8)	C26—H26	0.9500
C1—H1A	0.9800	C27—C28	1.357 (6)
C1—H1B	0.9800	C27—H27	0.9500
C1—H1C	0.9800	C28—C29	1.377 (5)
C2—C3^i^	1.521 (4)	C28—H28	0.9500
C2—C3	1.521 (4)	C29—H29	0.9500
C2—H2	1.0000	C30—O31	1.218 (5)
C3—C4	1.387 (5)	C30—H30	0.9500
C3—C8	1.397 (5)	O5—C32	1.360 (6)
C4—C5	1.388 (5)	O5—C40	1.439 (4)
C4—H4	0.9500	C32—C37	1.369 (7)
C5—C6	1.396 (4)	C32—C33	1.434 (11)
C5—C15	1.516 (5)	C33—C34	1.402 (6)
C6—C7	1.392 (5)	C33—C38	1.461 (7)
C7—C8	1.382 (5)	C34—C35	1.349 (9)
C7—C11	1.510 (5)	C34—H34	0.9500
C10—O1^i^	1.409 (4)	C35—C36	1.353 (8)
C10—H10A	0.9900	C35—H35	0.9500
C10—H10B	0.9900	C36—C37	1.380 (7)
C11—H11A	0.9900	C36—H36	0.9500
C11—H11B	0.9900	C37—H37	0.9500
C12—H12A	0.9900	C38—O39	1.205 (5)
C12—H12B	0.9900	C38—H38	0.9500
C13—C14	1.391 (5)	O5A—C32A	1.359 (16)
C13—C20	1.397 (5)	O5A—C40	1.439 (6)
C14—C17	1.383 (5)	C32A—C37A	1.367 (16)
C14—C15	1.516 (5)	C32A—C33A	1.43 (2)
C15—C16	1.528 (5)	C33A—C34A	1.413 (15)
C15—H15	1.0000	C33A—C38A	1.445 (16)
C16—H16A	0.9800	C34A—C35A	1.338 (17)
C16—H16B	0.9800	C34A—H34A	0.9500
C16—H16C	0.9800	C35A—C36A	1.354 (18)
C17—C18	1.393 (5)	C35A—H35A	0.9500
C17—H17	0.9500	C36A—C37A	1.383 (17)
C18—C19	1.386 (4)	C36A—H36A	0.9500
C18—C21	1.511 (4)	C37A—H37A	0.9500
C19—C20	1.393 (5)	C38A—O39A	1.147 (14)
C20—C40	1.483 (5)	C38A—H38A	0.9500
C21—C22	1.505 (7)	O39A—O39A^i^	0.81 (2)
C21—C18^i^	1.511 (4)	O39A—C38A^i^	1.769 (18)
C21—H21	1.0000	C40—H40A	0.9900
C22—H22A	0.9800	C40—H40B	0.9900
			
C8—O1—C10	115.9 (3)	C21—C22—H22A	109.5
C24—O2—C11	120.5 (3)	C21—C22—H22B	109.5
C6—O3—C12	116.8 (3)	H22A—C22—H22B	109.5
C13—O4—C12	115.8 (3)	C21—C22—H22C	109.5
C19—O6—C23	116.4 (3)	H22A—C22—H22C	109.5
C2—C1—H1A	109.5	H22B—C22—H22C	109.5
C2—C1—H1B	109.5	O6^i^—C23—O6	112.9 (4)
H1A—C1—H1B	109.5	O6^i^—C23—H23A	109.0
C2—C1—H1C	109.5	O6—C23—H23A	109.0
H1A—C1—H1C	109.5	O6^i^—C23—H23B	109.0
H1B—C1—H1C	109.5	O6—C23—H23B	109.0
C1—C2—C3^i^	113.9 (3)	H23A—C23—H23B	107.8
C1—C2—C3	113.9 (3)	O2—C24—C29	124.9 (4)
C3^i^—C2—C3	108.8 (4)	O2—C24—C25	114.8 (3)
C1—C2—H2	106.6	C29—C24—C25	120.2 (3)
C3^i^—C2—H2	106.6	C26—C25—C24	119.0 (4)
C3—C2—H2	106.6	C26—C25—C30	120.8 (4)
C4—C3—C8	116.7 (3)	C24—C25—C30	120.2 (4)
C4—C3—C2	122.2 (3)	C27—C26—C25	120.5 (4)
C8—C3—C2	121.0 (4)	C27—C26—H26	119.7
C3—C4—C5	123.0 (3)	C25—C26—H26	119.7
C3—C4—H4	118.5	C28—C27—C26	118.8 (4)
C5—C4—H4	118.5	C28—C27—H27	120.6
C4—C5—C6	118.1 (3)	C26—C27—H27	120.6
C4—C5—C15	121.9 (3)	C27—C28—C29	122.9 (4)
C6—C5—C15	119.8 (3)	C27—C28—H28	118.6
O3—C6—C7	118.1 (3)	C29—C28—H28	118.6
O3—C6—C5	120.8 (3)	C24—C29—C28	118.5 (4)
C7—C6—C5	121.1 (3)	C24—C29—H29	120.8
C8—C7—C6	118.4 (3)	C28—C29—H29	120.8
C8—C7—C11	122.2 (3)	O31—C30—C25	123.6 (4)
C6—C7—C11	119.4 (3)	O31—C30—H30	118.2
C7—C8—C3	122.8 (3)	C25—C30—H30	118.2
C7—C8—O1	117.7 (3)	C32—O5—C40	118.5 (5)
C3—C8—O1	119.4 (3)	O5—C32—C37	123.3 (8)
O1^i^—C10—O1	112.6 (4)	O5—C32—C33	117.8 (6)
O1^i^—C10—H10A	109.1	C37—C32—C33	118.6 (6)
O1—C10—H10A	109.1	C34—C33—C32	118.1 (4)
O1^i^—C10—H10B	109.1	C34—C33—C38	119.2 (4)
O1—C10—H10B	109.1	C32—C33—C38	122.7 (4)
H10A—C10—H10B	107.8	C35—C34—C33	121.1 (5)
O2—C11—C7	112.3 (3)	C35—C34—H34	119.4
O2—C11—H11A	109.1	C33—C34—H34	119.4
C7—C11—H11A	109.1	C34—C35—C36	120.1 (5)
O2—C11—H11B	109.1	C34—C35—H35	120.0
C7—C11—H11B	109.1	C36—C35—H35	120.0
H11A—C11—H11B	107.9	C35—C36—C37	121.7 (5)
O4—C12—O3	112.1 (3)	C35—C36—H36	119.1
O4—C12—H12A	109.2	C37—C36—H36	119.1
O3—C12—H12A	109.2	C32—C37—C36	120.0 (6)
O4—C12—H12B	109.2	C32—C37—H37	120.0
O3—C12—H12B	109.2	C36—C37—H37	120.0
H12A—C12—H12B	107.9	O39—C38—C33	124.3 (5)
O4—C13—C14	120.7 (3)	O39—C38—H38	117.9
O4—C13—C20	117.0 (3)	C33—C38—H38	117.9
C14—C13—C20	122.2 (3)	C32A—O5A—C40	107 (2)
C17—C14—C13	117.4 (3)	O5A—C32A—C37A	130.8 (19)
C17—C14—C15	122.3 (3)	O5A—C32A—C33A	108.2 (19)
C13—C14—C15	120.2 (3)	C37A—C32A—C33A	118 (2)
C5—C15—C14	108.9 (3)	C34A—C33A—C32A	116.4 (15)
C5—C15—C16	113.8 (3)	C34A—C33A—C38A	121.3 (14)
C14—C15—C16	114.4 (3)	C32A—C33A—C38A	121.3 (14)
C5—C15—H15	106.4	C35A—C34A—C33A	120.6 (16)
C14—C15—H15	106.4	C35A—C34A—H34A	119.7
C16—C15—H15	106.4	C33A—C34A—H34A	119.7
C15—C16—H16A	109.5	C34A—C35A—C36A	120.7 (19)
C15—C16—H16B	109.5	C34A—C35A—H35A	119.7
H16A—C16—H16B	109.5	C36A—C35A—H35A	119.7
C15—C16—H16C	109.5	C35A—C36A—C37A	121.2 (19)
H16A—C16—H16C	109.5	C35A—C36A—H36A	119.4
H16B—C16—H16C	109.5	C37A—C36A—H36A	119.4
C14—C17—C18	123.0 (3)	C32A—C37A—C36A	118.3 (19)
C14—C17—H17	118.5	C32A—C37A—H37A	120.9
C18—C17—H17	118.5	C36A—C37A—H37A	120.9
C19—C18—C17	117.4 (3)	O39A—C38A—C33A	121.4 (15)
C19—C18—C21	120.8 (3)	O39A—C38A—H38A	119.3
C17—C18—C21	121.8 (3)	C33A—C38A—H38A	119.3
C18—C19—O6	120.1 (3)	O39A^i^—O39A—C38A	128.8 (10)
C18—C19—C20	122.4 (3)	C38A—O39A—C38A^i^	98.4 (15)
O6—C19—C20	117.3 (3)	O5A—C40—C20	103.8 (10)
C19—C20—C13	117.6 (3)	O5—C40—C20	109.1 (3)
C19—C20—C40	121.0 (3)	O5A—C40—H40A	107.3
C13—C20—C40	121.4 (3)	O5—C40—H40A	109.9
C22—C21—C18^i^	115.1 (3)	C20—C40—H40A	109.9
C22—C21—C18	115.1 (3)	O5A—C40—H40B	117.5
C18^i^—C21—C18	107.4 (3)	O5—C40—H40B	109.9
C22—C21—H21	106.2	C20—C40—H40B	109.9
C18^i^—C21—H21	106.2	H40A—C40—H40B	108.3
C18—C21—H21	106.2		
			
C1—C2—C3—C4	35.0 (5)	C14—C13—C20—C19	2.5 (5)
C3^i^—C2—C3—C4	−93.2 (4)	O4—C13—C20—C40	0.3 (4)
C1—C2—C3—C8	−148.0 (4)	C14—C13—C20—C40	−175.3 (3)
C3^i^—C2—C3—C8	83.8 (5)	C19—C18—C21—C22	146.8 (4)
C8—C3—C4—C5	0.0 (5)	C17—C18—C21—C22	−36.1 (5)
C2—C3—C4—C5	177.2 (3)	C19—C18—C21—C18^i^	−83.7 (4)
C3—C4—C5—C6	−1.6 (5)	C17—C18—C21—C18^i^	93.4 (4)
C3—C4—C5—C15	−176.1 (3)	C19—O6—C23—O6^i^	−92.0 (4)
C12—O3—C6—C7	−101.0 (3)	C11—O2—C24—C29	−24.0 (5)
C12—O3—C6—C5	81.8 (4)	C11—O2—C24—C25	159.5 (3)
C4—C5—C6—O3	179.5 (3)	O2—C24—C25—C26	173.7 (3)
C15—C5—C6—O3	−5.9 (5)	C29—C24—C25—C26	−3.0 (5)
C4—C5—C6—C7	2.4 (5)	O2—C24—C25—C30	−7.6 (5)
C15—C5—C6—C7	177.0 (3)	C29—C24—C25—C30	175.6 (3)
O3—C6—C7—C8	−178.8 (3)	C24—C25—C26—C27	2.1 (5)
C5—C6—C7—C8	−1.7 (5)	C30—C25—C26—C27	−176.5 (4)
O3—C6—C7—C11	3.3 (5)	C25—C26—C27—C28	−0.4 (6)
C5—C6—C7—C11	−179.6 (3)	C26—C27—C28—C29	−0.6 (7)
C6—C7—C8—C3	0.0 (5)	O2—C24—C29—C28	−174.3 (3)
C11—C7—C8—C3	177.9 (3)	C25—C24—C29—C28	2.1 (5)
C6—C7—C8—O1	178.8 (3)	C27—C28—C29—C24	−0.2 (6)
C11—C7—C8—O1	−3.3 (5)	C26—C25—C30—O31	−1.8 (6)
C4—C3—C8—C7	0.8 (5)	C24—C25—C30—O31	179.6 (4)
C2—C3—C8—C7	−176.4 (3)	C40—O5—C32—C37	11.7 (17)
C4—C3—C8—O1	−178.0 (3)	C40—O5—C32—C33	−162.8 (8)
C2—C3—C8—O1	4.8 (5)	O5—C32—C33—C34	−178.7 (8)
C10—O1—C8—C7	99.5 (4)	C37—C32—C33—C34	6.6 (12)
C10—O1—C8—C3	−81.7 (4)	O5—C32—C33—C38	0.3 (13)
C8—O1—C10—O1^i^	95.5 (4)	C37—C32—C33—C38	−174.4 (7)
C24—O2—C11—C7	−74.8 (4)	C32—C33—C34—C35	−3.8 (10)
C8—C7—C11—O2	119.6 (4)	C38—C33—C34—C35	177.3 (6)
C6—C7—C11—O2	−62.6 (4)	C33—C34—C35—C36	0.9 (11)
C13—O4—C12—O3	93.9 (4)	C34—C35—C36—C37	−0.8 (11)
C6—O3—C12—O4	−93.4 (3)	O5—C32—C37—C36	178.9 (9)
C12—O4—C13—C14	−82.4 (4)	C33—C32—C37—C36	−6.7 (13)
C12—O4—C13—C20	101.9 (3)	C35—C36—C37—C32	3.9 (11)
O4—C13—C14—C17	−177.5 (3)	C34—C33—C38—O39	−2.2 (8)
C20—C13—C14—C17	−2.1 (4)	C32—C33—C38—O39	178.9 (7)
O4—C13—C14—C15	4.9 (4)	C40—O5A—C32A—C37A	−18 (8)
C20—C13—C14—C15	−179.7 (3)	C40—O5A—C32A—C33A	142 (4)
C4—C5—C15—C14	91.1 (3)	O5A—C32A—C33A—C34A	173 (3)
C6—C5—C15—C14	−83.3 (4)	C37A—C32A—C33A—C34A	−24 (6)
C4—C5—C15—C16	−37.8 (5)	O5A—C32A—C33A—C38A	4(6)
C6—C5—C15—C16	147.7 (3)	C37A—C32A—C33A—C38A	167 (3)
C17—C14—C15—C5	−92.5 (3)	C32A—C33A—C34A—C35A	12 (5)
C13—C14—C15—C5	85.0 (4)	C38A—C33A—C34A—C35A	−179 (3)
C17—C14—C15—C16	36.0 (5)	C33A—C34A—C35A—C36A	−1(5)
C13—C14—C15—C16	−146.5 (3)	C34A—C35A—C36A—C37A	0(6)
C13—C14—C17—C18	1.1 (4)	O5A—C32A—C37A—C36A	−177 (6)
C15—C14—C17—C18	178.7 (3)	C33A—C32A—C37A—C36A	24 (7)
C14—C17—C18—C19	−0.6 (4)	C35A—C36A—C37A—C32A	−13 (5)
C14—C17—C18—C21	−177.8 (3)	C34A—C33A—C38A—O39A	−1(3)
C17—C18—C19—O6	176.4 (3)	C32A—C33A—C38A—O39A	168 (4)
C21—C18—C19—O6	−6.4 (4)	C33A—C38A—O39A—O39A^i^	141.6 (14)
C17—C18—C19—C20	1.0 (4)	C33A—C38A—O39A—C38A^i^	141.6 (14)
C21—C18—C19—C20	178.3 (3)	C32A—O5A—C40—O5	−45 (13)
C23—O6—C19—C18	83.0 (4)	C32A—O5A—C40—C20	−179 (4)
C23—O6—C19—C20	−101.4 (4)	C32—O5—C40—O5A	130 (15)
C18—C19—C20—C13	−1.9 (5)	C32—O5—C40—C20	178.1 (9)
O6—C19—C20—C13	−177.4 (3)	C19—C20—C40—O5A	104.3 (19)
C18—C19—C20—C40	175.8 (3)	C13—C20—C40—O5A	−78.1 (19)
O6—C19—C20—C40	0.3 (4)	C19—C20—C40—O5	98.3 (6)
O4—C13—C20—C19	178.0 (3)	C13—C20—C40—O5	−84.1 (6)

Symmetry codes: (i) *x*, −*y*+1/2, *z*.
